# Use of Methylcellulose-Based Pellet to Enhance the Bacterial Self-Healing of Cement Composite

**DOI:** 10.3390/ma14206113

**Published:** 2021-10-15

**Authors:** Indong Jang, Dasom Son, Yongjun Son, Jihyeon Min, Chongku Yi

**Affiliations:** 1 Construction Material Laboratory, School of Civil, Environmental and Architectural Engineering, Korea University, Seoul 02841, Korea; indongjang7761@korea.ac.kr (I.J.); da5som@korea.ac.kr (D.S.); 2 Laboratory of Molecular Environmental Microbiology, Department of Environmental Science and Ecological Engineering, Korea University, Seoul 02841, Korea; 2020010812@korea.ac.kr (Y.S.); m1nzaa@korea.ac.kr (J.M.)

**Keywords:** methylcellulose, bacteria, self-healing concrete

## Abstract

In this study, a new type of bacterial carrier using methylcellulose was presented, and its applicability to self-healing concrete has been explored. Methylcellulose, the main component of a 2 mm pellet-shaped carrier, can remain stable in alkaline environments and expand in neutral or acidic environments. These properties allow bacteria to survive in the high-alkaline and high-pressure environments of early age concrete, and the number of bacteria increases rapidly in the event of cracks, accelerating crack closure. The results show that the survival rate of bacterial spores inside the mortar was increased, and the pellet provides an enhanced biological anchor suitable for bacterial activity, bacterial growth, and mineral precipitation. Further, the results indicate an improved self-healing efficiency compared with mixing bacteria directly into the cement composite.

## 1. Introduction

The occurrence of micro- to macro-crack propagation increases the probability of the entry of substances into concrete, thereby reducing the durability of the material and the overall durability of the built structure [1]. Such propagation can be inhibited by self-healing processes of cementitious materials, specifically the autogenous healing mechanism [2], which is an intrinsic repair property of cementitious material. Autogenous healing can be accelerated by applying mineral admixture [3], fiber [4,5], nanofiller [6], and curing agent [7,8]. Another self-healing concrete process is the autonomous healing, which involves the incorporation of capsules containing healing agents or bacteria spores to the composites including electrodeposition [9,10], epoxy injection [11,12,13], polymers [13,14,15], and shape-memory alloys [16].

The application of bacteria as a self-healing technique has great potential and effectivity by successfully filling cracks with calcium carbonate precipitation through biomineralization [17,18,19,20,21,22,23,24,25,26,27,28,29,30,31,32,33]. The method of microbially induced calcium carbonate precipitation (MICP) is advantageous over other mechanisms because of the following reasons: (1) it prevents the depletion of healing materials since bacteria can produce minerals by consuming nutrients from the ambient environment [23] and (2) the self-healing validity of bacteria without efficiency degradation is secured due to its ability to survive in extreme conditions and last for a few decades [34].

MICP is a complex biochemical process affected by two conditions: the survival rate of the associated bacterial species through long curing time [17,28] and the environmental state of the system [35], which is determined by various factors such as calcium ion concentration, pH level, dissolved inorganic carbon (DIC), and nucleation site availability. In normal concrete, the pH level of its environment reaches 12 or higher due to the presence of hydroxide ions (OH^−^) [36], which presents a low available oxygen concentration for the bacteria. Moreover, dehydrating conditions and high mechanical compressive forces during curing are also experienced within the concrete. With such harsh conditions, alkali-tolerant and spore-forming bacteria species (i.e., Gram-positive Bacillus species) are suitable for application in self-healing concrete and are being studied intensively [17]. It has been discovered that the bacterial cell wall and extracellular polymeric substance (EPS) provide nucleation sites during the microbial carbonate precipitation in the presence of available Ca^2+^ ions, and the metabolic activity efficiency of the bacteria is driven by DIC and is surrounded by an alkaline environment (high pH and increase in DIC) [37]. 

In a previous study, multibacterial spores from alkaliphilic and alkali-tolerant strains were spray-dried into cementitious material, and they enhanced the bacteria’s survival and crystalline formation and reduced the cement mortar’s sorptivity, which is related to the pore system of the cement [38]. On the other hand, several studies have reported the use of carriers including diatomaceous earth [24,39] and lightweight aggregates [25,40,41] to improve the MICP self-healing capability, because the direct application of bacteria in a high-pH environment subject to hydration process can decrease their activity and survival rate. Some studies also suggested the use of bacterial encapsulation in various materials such as silica gel [33], hydrogel [18], polyurethane [33,39], melamine microcapsules [30], and graphite nanoplatelets [40], which show that encapsulated bacteria cement composites are superior in crack healing compared to unencapsulated ones.

Methylcellulose (MC) is a methyl ester containing 27–31% methoxy groups, commonly used as a thickener, emulsifier, and coating agent, which is characterized by its nontoxicity and high adhesion [41]. One of the distinctive characteristics of polymerized MC is that its polymer matrix swells in weakly acidic (down to pH 5) or neutral conditions but remains stable in alkaline solutions [42]. This shows the potential of MC as a great bacterial carrier since it can protect its contents from the high alkali environment of cement composites, and at the same time, it can release (i.e., by matrix swelling) the bacteria and other nutrients once cracks cause an inflow of water and decrease the surrounding pH.

In this study, a novel bacterial carrier using methylcellulose was proposed. Improved self-healing capacity of cement composites using MC as a carrier in the form of 2 mm-diameter pellets, including cocultured spray-dried bacteria, was investigated. The pellet consists of three components: methylcellulose polymer as a binder, SiO_2_ or diatomaceous earth as filler, and bacteria/nutrients as active substances. A mixture of pellet components was extruded using a hydraulic system and shaped into a short cylinder. Pellets will be used as a substitute for fine aggregate in the application stage, so it was extruded to a thickness of 2 mm through a preliminary experiment. Cocultured *Lysinibacillus boronitolerans* YS11 and *Bacillus* sp. AK13 was used in the previous research [38] and were incorporated with the cement mortar. The degree of expansion of the bacterial pellets in alkaline and neutral solution was first investigated to prove the concept of MC explained the earlier paragraph. In addition, the difference in bacterial activity on the surface of the mortar caused by the pellets was confirmed by a bacterial fluorescence staining technique. Bacterial protection by the pellet against the hydration of the cement composite was measured using CFU technique for 28 days, and the actual crack healing performance for 0.35 mm or smaller cracks was confirmed through the water permeability test.

## 2. Materials and Methods

### 2.1. Bacteria Preparation

In this study, the cocultured spray-dried bacteria powder proposed in the previous study was used [38]. The cocultured spray-dried bacteria powder was produced through the following steps: (1) culturing the two living bacteria in Luria–Bertani (LB) medium, (2) inoculating both bacteria to the spore-producing mass medium, and (3) drying it through a spray dryer.

*L. boronitolerans* YS11 [43] and *Bacillus* sp. AK13 [44] strains were inoculated separately in 5 mL LB medium in test tubes. The LB medium consisted of 0.5% yeast extract (Bioshop), 1% tryptone (Bioshop), and 1% sodium chloride (Sigma-Aldrich). Its pH was then adjusted to 8 using 1 M NaOH. The bacteria-inoculated LB medium was incubated in an orbital shaker at 37 °C and 220 rpm for 24 h. 

The samples, 1 × 10^6^ CFU/mL YS11 and 3 × 10^6^ CFU/mL AK13, were inoculated in 100 mL of pH 8 Difco Sporulation Medium (DSM). The DSM consisted of 8 g/L nutrient broth (Difco), 1 g/L KCl, and 0.25 g/L MgSO_4_, and its pH was adjusted to 8 using 1 M NaOH. The pH-adjusted solution was then autoclaved and mixed with 164 mg/L Ca(NO_3_)_2_, 1.26 mg/L MnCl_2_, and 0.15 mg/L FeSO_4_, which were filtered using a 0.25 µm syringe filter. For the mass culture, 100 mL of bacteria was inoculated in 8 L of DSM in a 10 L scale bioreactor (BIOCNS), followed by incubation at 37 °C, 2 L/min airflow, and 100 rpm for 48 h.

The DSM was spray-dried by using a spray dryer SD-Basic (LabPlant) operated at 165 °C inlet temperature, 45% of maximum pump speed (13.3 mL/min), and 1.8 MPa airflow volume, while the outlet temperature was maintained at 80 ± 2 °C. The cocultured bacterial powder produced by spray drying is a yellow-colored particulate.

### 2.2. Methylcellulose-Based Pellet Materials

The components needed for the MC-based pellet and their mixing ratios are listed in Table 1. MC is a pH-dependent expansive binder, as stated in the introduction. Microcrystalline cellulose (MCC) is also used as a binder with a relatively low ratio compared with MC but it enhances the particle’s strength/hardness due to its fibrous structure. The diatomaceous earth, which is mostly composed of SiO_2_, has showcased its large surface area due to the presence of fossilized hard-shelled diatoms. DE absorbs bacteria and nutrients on its surface, while SiO_2_ acts as an inert filler. The total amount of DE absorbed content is 0.5 in weight ratio compared to the MC content.

### 2.3. Methylcellulose-Based Pellet Fabrication Process

Empty pellet (EP) and bacterial pellet (BP) were fabricated in this study. BP contains spray-dried cocultured bacteria and nutrients, while EP only contains nutrients.

The pellet fabrication process consisted of four major steps: (1) mixing components’ constituents listed in Table 1 into a thick paste with proper consistency, (2) extrusion of the mixture into approximately 2 mm diameter with a cylindrical shape using a hydraulic pump, (3) cutting the cylinder-shaped mixture with a 2–4 mm-length dimension, and (4) drying in the oven. 

The methylcellulose was polymerized with water using a mortar mixer. In the preliminary experiments, around 4 times the weight of water to MC weight was needed to complete the polymerization. After adequate mixing, MCC and SiO_2_ were added to the polymer. Then, the DE, bacteria, calcium lactate, and yeast extract were dry mixed until the mixture retained a uniform concentration and moisture content. By mixing the dry compounds and the polymer with the mechanical mixer, a thick paste for MC-based pellet production was obtained.

**Figure 1:**
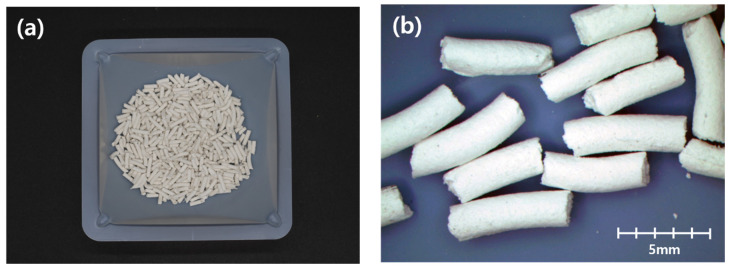
Methylcellulose-based pellet: (**a**) samples of manufactured pellet and (**b**) 20× magnified image.

Strands of pellets were produced through hydraulic press extrusion with a thickness of 2 mm, which was the smallest diameter the machine could extrude due to viscosity of the pellet paste and the capacity of the hydraulic press. Then, these strands were oven dried at 60 °C for more than three days until no change in weight was observed. After drying, strands were cut into 4–8 mm-long pellets and stored in an RH 50% condition. Figure 1 shows the methylcellulose-based pellet.

### 2.4. Mortar Preparation

Type 1 Portland cement (Grade 43, Korea), ISO standard sand (ISO 679: 2009), and tap water were used to prepare the mortar. The mixing proportions are summarized in Table 2. Mortar mixing was performed according to the ASTM C349 standard. The reference mortar (RM) refers to a plain mortar without additional materials, and the bacterial mortar (BM) included a mixture of bacterial spore powder described in Section 2.1, their nutrients, calcium lactate, and yeast extract. The bacterial mixture consisted of about 2 times the yeast extract and 4 times the calcium lactate per unit mass compared with the cocultured bacterial spore powder. Both EPM (Empty pellet mortar) and BPM (Bacterial pellet mortar) mortars contain 5% of the weight of cement for EP and BP. The target bacteria concentration in BM mortar was set at 5 × 10^7^ cell/mL of mortar for comparison with other studies. In the case of BPM, 5% incorporation was determined through prior research which did not significantly affect the compressive strength and flow of cement mortar. Bacterial concentration of BPM was 1.8 × 10^7^ cell/1 mL mortar, which is around 35% of the bacterial concentration of BM.

When observed with the naked eye after the mixing, no damage to the pellets was found. In addition, since pellets remained as pellets after the damage, broken pellets produced during mortar mixing do not significantly affect the performance of the pellets.

### 2.5. Pellet Volume Expansion in Different pH Solutions

To observe the pellet behavior under alkaline conditions, the pellet was immersed in a solution consisting of distilled water, 1 M NaOH, and saturated Ca(OH)_2_. The pellet diameter after immersion in the solution (d_m_) was measured in the plane direction every time interval of 0 min, 15 min, 1 h, 3 h, 12 h, and 24 h. The expansion of the pellet (E_m_) and the expansion rate per minute (R_m,n_) were calculated using Equations (1) and (2), respectively.

(1) $$E_{m}=\frac{d_{m}-d_{0}\;}{d_{0}}\times 100\left(\%\right)$$

(2) $$R_{m,n}=\frac{d_{m}-d_{n}\;}{m-n}\left(\mathrm{mm}/\min\right)$$

where d_m_ is the pellet diameter(mm) after immersion in the solution, m and n is immersion time interval in minute.

### 2.6. Bacterial Activity over Mortar Surface and XRD Analysis

Bacterial activity over the mortar surface was observed using the SYTO^®^ 9 green, fluorescent dye. This technique works by staining nucleic acids (DNA and RNA) of Gram-positive live bacteria, then absorbing the light with wavelengths ranging from 450 to 530 nm. This produces a green, fluorescent light emission. Then, 5 mM fluorescent dye was diluted to 50 µM using phosphate-buffered saline (PBS).

RM, BM, and BPM were casted in a cylindrical mold with a diameter and a height of 3 cm. After demolding, all specimens were immersed in tap water at room temperature for seven days. A 5 mm portion in the middle of the mortar cylinders was sliced using a low-speed saw with a 10.16 cm diamond wafering blade (Buehler). The sliced mortars were water-cured in room temperature separately for three days.

Finally, 20 μL of diluted fluorescent dye was spread over one side of the sliced mortars in the dark room. Specimens were mounted on an inverted microscope for epifluorescence (Carl Zeiss, Axiovert 200M), and the exposure time was set to 0.1 s.

After bacterial activity observations, BM and BPM specimens were dried in an oven at 105 °C for one day to remove the extracellular polymeric substance over the specimen surface. The surface of the specimen, once cooled to room temperature, was scratched with a sharp-edged tool to collect crystalline, and the crystals were crushed into particulates using a mortar and pestle. XRD analysis of powdered crystalline was performed using D/MAX-2500 (Rigaku, Japan) from 5 to 80 2-theta range, in 40 kV, 200 mA.

### 2.7. Bacterial Viability in Cement Mortar

The colony-forming unit (CFU) technique [45] was used for bacterial viability quantification [38]. The centers of the RM, BM, and BPM samples were ground into a fine powder using a hammer mill. Ground particles (0.5 g) were agitated in 10 mL of PBS using a vortex mixer for 10 min to separate the bacteria from the mortar. Each mortar sample solution was serially diluted 10^−1^ to 10^−6^ with PBS. Diluted solutions were placed on 3 M Petrifilm (for aerobic counting) and stored at 35 °C and 50% RH. Red dots in the plate correspond to the bacterial colonies. Based on the dilution rate, the number of bacteria in 1 g of each mortar sample was calculated using the red dot counts over the films. The CFU test was performed for curing ages of 1, 3, 7, 14, and 28 days. 

### 2.8. Mortar Flow Test and Compressive Strength

Immediately after the mixing of RM, BM, EPM, and BPM mortar, a flow test was carried out in accordance with ASTM C1437. Immediately after mixing, the flow was recorded six times on a 10 min basis for a total of 1 h. The compressive strengths of RM, BM, EPM, and BPM at curing times of 3, 7, 14, and 28 days were measured according to KS L ISO 679 using 40 mm × 40 mm × 160 mm prisms.

### 2.9. Water Permeability of Crack-Induced Mortar

**Figure 2:**
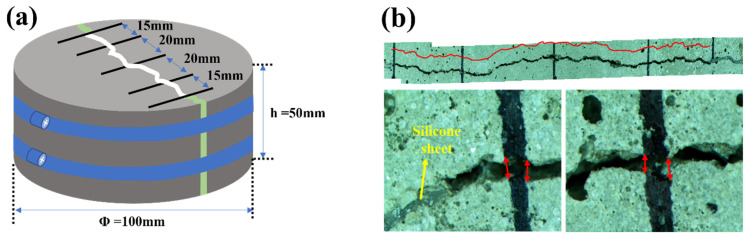
Crack-induced specimen for water permeability test: (**a**) schematic of the crack-induced specimen; (**b**) example of representative length and crack measurement of specimen.

The water permeability test was conducted according to Shin et al. [46]. First, each RM, BM, EPM, and BPM were casted in an acrylic mold with 50 mm height and 100 mm diameter. Then, they were cured in tap water for 14 days, and were split into half using a concrete splitting tensile-strength test setup. A silicone sheet with a thickness of 0.3 mm and a width of 15 mm was inserted at each end of the split mortar specimens and was secured with a hose band. As shown in Figure 2a, 5-line markers perpendicular to the crack are drawn. The crack length (C_L_) is the total crack length, except for the silicone sheet inserted into the specimen, and the average crack width (C_w_) was obtained by averaging 10 measurements of the distance between the marker passing over the crack shown in Figure 2b. Afterwards, the specimens were water cured at 23 °C until the experiment was performed.

**Figure 3:**
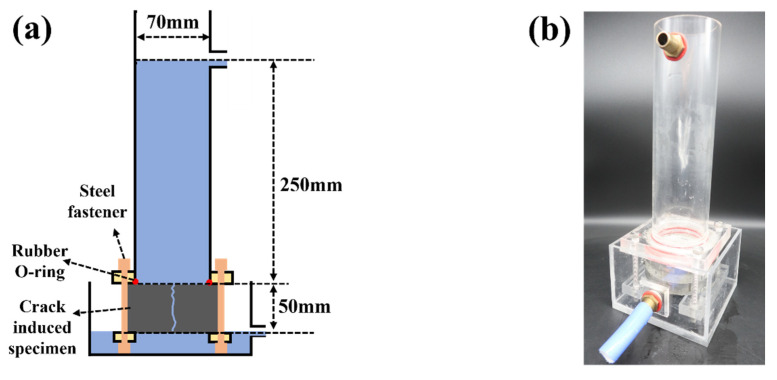
Water permeability test setup: (**a**) schematic of water permeability test setup; (**b**) picture of test setup.

The specimens were loaded into the device shown in Figure 3, maintaining a water height of 300 mm with a 3 min wait to stabilize the water spillage through the crack. The quantity of spillage on the curing day was measured for the next 10 min. The average water flow permeability per unit crack length and minute (Q_t_, mL/mm∙min) was determined. The crack healing rate is defined as follows:

(3) $$\mathrm{healing}\;\mathrm{rate}\left(\%\right)=\frac{\left(Q_{0}-Q_{t}\right)}{Q_{0}}\times 100$$

where Q_0_ is the initial spillage per unit crack length and minute measurement immediately after the crack specimen was made and Q_t_ was measured on days 7, 14, 21, and 28. Expect for the permeability test, all specimens were cured completely submerged in 25 °C water.

## 3. Results

### 3.1. Pellet Behavior in Different pH Solutions

**Figure 4:**
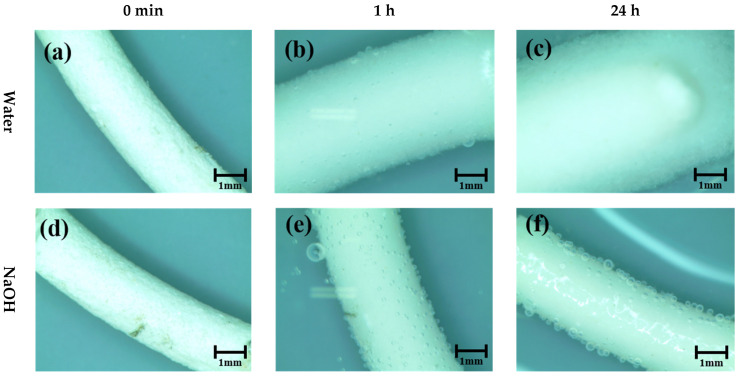

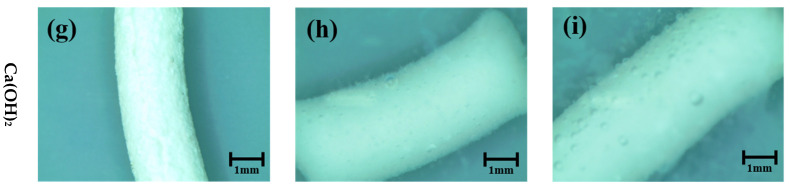
Pellet expansion behavior in water (**a**–**c**), 1 M NaOH (**d**–**f**), and saturated Ca(OH)2 solution (**g**–**i**).

**Figure 5:**
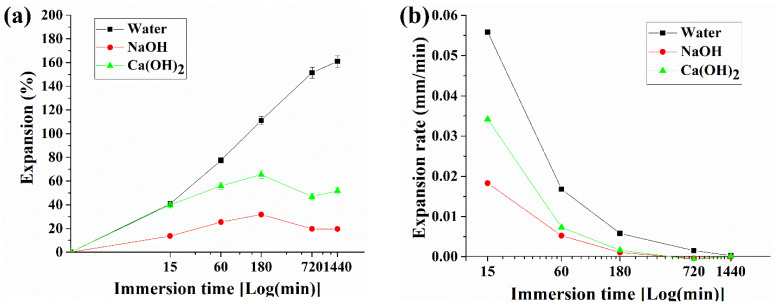
Expansion (**a**) and expansion rate through immersion time (**b**). The x-axis is time, expressed as the log of minutes.

Figure 4 illustrates the pellet expansion in water, 1 M NaOH, and saturated Ca(OH)_2_ immediately after input (0), 1, and 24 h. On the other hand, Figure 5 shows the changes in pellet expansion (Em) and expansion rate (Rm) calculated from Equations (1) and (2), respectively, through observations at 0 and 15 min and 1, 3, 12, and 24 h.

The pellet before expansion maintains an average thickness of 2.11 mm (SD 0.036 mm) and begins to expand by contacting with each testing liquid. The pellets in contact with water expanded to an average of 3.6 mm (SD 0.091 mm, a 77.4% increase from the original pellet’s thickness) within 1 h, and an average of 5.3 mm (SD 0.151 mm, 161.0%) within 24 h. However, the increase in size of the pellets immersed in NaOH solution was 2.52 mm (SD 0.109 mm, 25.4%) in 1 h and 2.41 mm (SD 0.095 mm, 19.5%) in 24 h. The pellets submerged in the Ca(OH)_2_ solution expanded to 3.18 mm (SD 0.031 mm, 55.9%) in 1 h and 3.09 mm (SD 0.037 mm, 51.8%) in 24 h.

In terms of the expansion rate, the initial 15-min expansion rate in water was 0.559 mm/min, after which it reached 0.0168 mm/min in 1 h and decreased to 0.00027 mm/min in 24 h. In the NaOH solution, the expansion rate of the pellets was maintained at 1/5 of that of water. Then, the rate reached 0.018 mm/min in 15 min and 0.001 mm/min in 1 h. Thereafter, the pellets did not expand or show any tendency of shrinking. Thus, the experimental results showed −0.0045 mm/min at 12 h and −0.000002 mm/min at 24 h. 

As described in the introduction, MC, the main component of pellets, expands in weak acidity and neutral solutions, and remain stable in alkali solutions. As a result of the experiment, although there is some swelling in the neutral solution (water), the stronger the basic solution, the greater the expansion of the pellet, and it was confirmed that the behavior according to the pH of MC works as described in the introduction. The small decreasing trend after 180 min for NaOH and Ca(OH)_2_ types in Figure 5a is assumed to indicate a stabilization phase of pellet in the alkaline solution

### 3.2. Bacterial Activity over Mortar Surface

**Figure 6:**
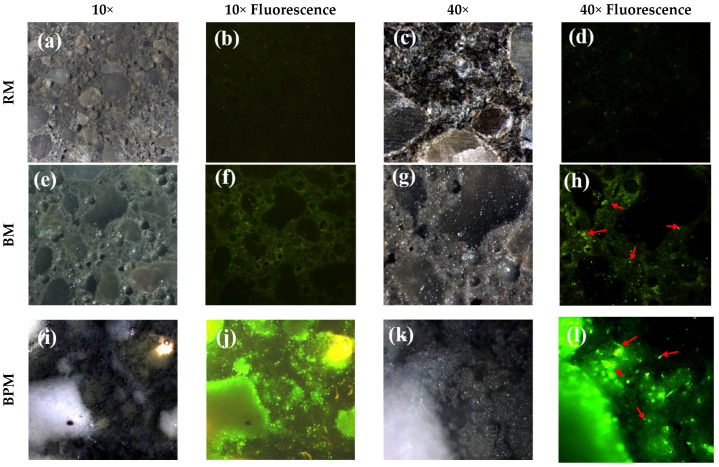
Observation of fluorescence-dyed bacteria distribution over RM (**a**–**d**), BM (**e**–**h**), and BPM (**i**–**l**). Halogen lamp and fluorescence photographing were performed in an environment of 10× and 40× magnification, and the exposure time was set equal to 0.1 s. Red arrows indicate the bacteria colony.

Figure 6 shows the distribution of bacteria in the mortar specimens by fluorescence staining. The staining solution stains the DNA and RNA of living bacteria, absorbs light at wavelengths of 485–486 nm, and emits light wavelengths of 498–501 nm. In the case of fluorescence images, an exposure time of 0.1 s was set to facilitate a proper comparison of the bacterial distribution based on bacterial fluorescence.

In the case of the RM specimen (Figure 6a–c), neither crystal formation nor bacteria can be observed. A faintly visible green light is discerned by the fluorescence emitted by the fluorescent substance itself without binding to bacteria. However, a small number of bacteria can be observed in Figure 6d due to the biological contamination observed at 40× magnification. On the contrary, green light can be clearly identified in the area excluding the aggregate at 10× magnification in Figure 6f,h. In addition, in the case of BM, thin crystals are formed on the paste and aggregate at 40× magnification.

In the case of BPM, crystals covered the surface, making it impossible to distinguish between the aggregate and paste. The bright white material in Figure 6i is a pellet around which bacterial clusters can be observed, as shown in Figure 6j. Moreover, at 40× magnification, transparent crystals are significantly distributed on the BPM surface compared to both BM and RM, which are surrounded by bacterial colonies.

**Figure 7:**
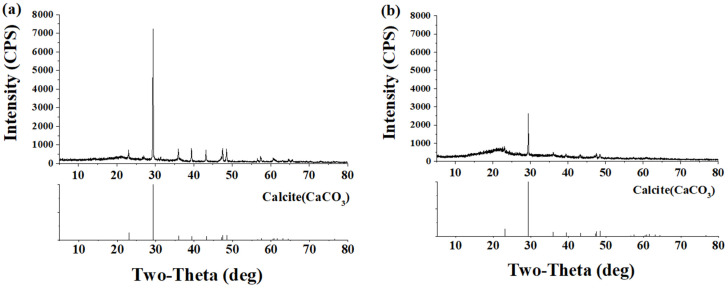
XRD result of collected crystalline over surface of BPM (**a**) and BM (**b**) specimen.

The results of the XRD analysis by collecting surface crystals of BM and BPM specimens are shown in Figure 7, and calcite formation over the surface was confirmed.

### 3.3. Bacterial Viability in Cement Mortar

**Figure 8:**
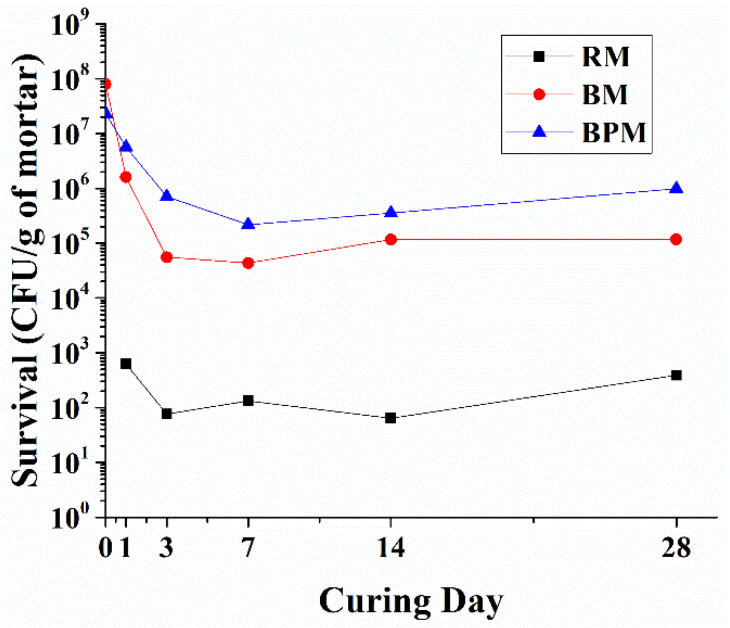
Survival rate of the bacteria calculated from CFU to the 1 g of ground mortar specimens from day 0 to day 28. The values of day 0 for RM, BM and BPM are calculated on the value diluted in the mortar based on the bacteria density measured just before mortar incorporation.

Figure 8 shows the number of bacteria surviving inside the cement mortar after 28 days of curing, with the y-axis representing the logarithm of the survival counts of bacteria per gram of mortar and the x-axis representing the 1st, 3rd, 7th, 14th, and 28th days of CFU measurement after curing. 

RM is the control group and contains the cases where the CFU was counted due to biological contamination from materials such as cement, sand, and tap water or during the curing ages. In this case, bacterial survival, or biological contamination, was maintained at an average of 250 cell/1 g of mortar throughout the curing period.

The initial incorporation of bacteria for BM is approximately 3.5 times higher than in BPM. Compared to the initial incorporation, the survival rate of bacteria in BM decreased to 2.0% on curing day 1 to the input bacteria number, 0.7% on day 3, and remained at an average of 0.115% until the 28th day. Unlike in BM, which maintains a flat state from day 3, BPM shows a moderate reduction in survival rate until day 7, when the flat curve appears. The survival of the BPM case decreased to 24.6% on the 1st day, 3.1% on the 3rd day, 0.9% on the 7th day, and maintained an average of 2.26% until the 28th curing age to the bacteria input.

### 3.4. Compressive Strength and Flow 

**Figure 9:**
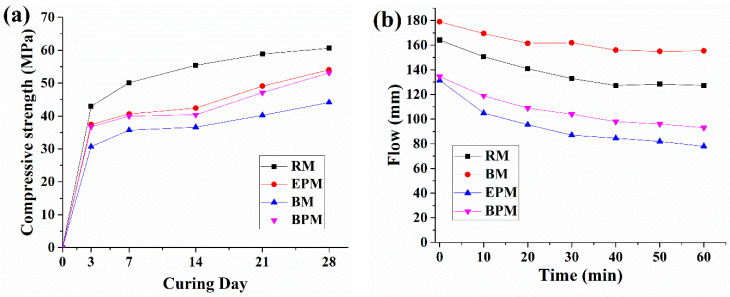
Compressive strength (**a**) and flow (**b**) of mortar specimens at curing days 3, 7, 14, 21 and 28.

Figure 9a shows the strength development of the mortar specimens with curing age. The RM shows a strength of 42.9 MPa on curing day 3 and reaches up to 60.6 MPa at day 28. The cocultured bacteria resulted in a 30.0% reduction in the average strength of the RM throughout the curing period. EPM and BPM showed similar compressive strengths over the entire curing day. Their strength reductions were 10.9% and 12.38% for EPM and BPM, respectively, on day 28. 

Figure 9b shows the results of the flow tests. The flow of the RM specimen measured immediately after mixing was 164 mm, and then decreased linearly to 127 mm by 40 min. The RM maintained at a flow of 127 mm for 60 min. For BM specimens, the initial flow (0 min) was approximately 15 mm higher than that of RM, and then, similar to the RM, the flow reduced linearly by 40 min to 155 mm and then maintained for 60 min. In contrast to BM, both the EPM and the BPM showed a steady decrease in the flow value until 60 min of measurement time, starting at 131 mm and 134 mm each, which is approximately 80% of that of the BM. After 60 min of measurements, the EPM and BPM were 78 mm and 93 mm, respectively.

### 3.5. Water Permeability of Crack-Induced Mortar

**Figure 10:**
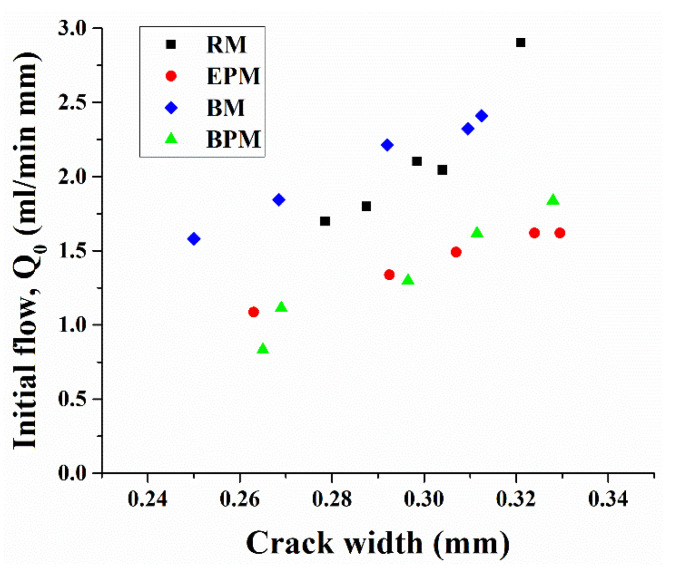
Initial water flow rate versus crack width.

**Figure 11:**
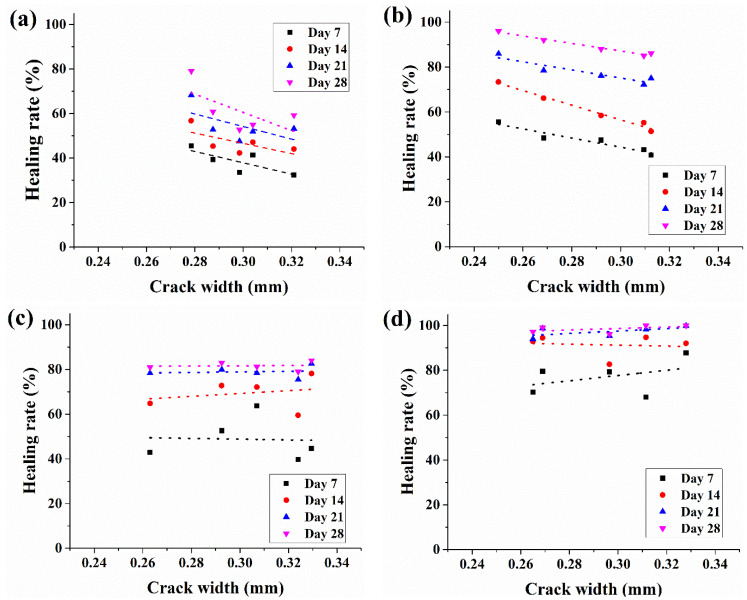
Water permeability through crack-induced mortar specimen: (**a**) RM, (**b**) BM, (**c**) EPM and (**d**) BPM.

The initial water flow through the crack specimens and the crack-healing rate from the water permeability test are shown in Figure 10 and Figure 11. For each crack-induced specimen, crack sizes in the range of 0.25–0.34 mm have been measured. As shown in Figure 10, the relationship between the crack width and initial water flow tends to increase as the crack size increases, which can be explained by Poiseuille’s Law which states that the water permeability through a small duct is proportional to the third power of the crack width. However, in the case of RM and BM, the initial water permeability at a crack size of 0.3 mm was 2.16 and 2.23 mL/min·mm, respectively, whereas EPM and BPM showed lower water flow at 1.42 and 1.4 mL/min·mm.

Figure 11 shows the changes in water flow (healing rate) of each specimen measured weekly over a month. Each dot in the same crack width represents the reduced water flow rate through the specimen crack calculated from Equation (3). Dotted trend lines show regressed healing rates of five specimens of curing days from week one to week four. 

The four graphs in Figure 11 can be classified according to the characteristics of the regression line. First, in Figure 11a,b, the regression lines are oriented downward, and the variance is not severe, whereas in Figure 11c,d, the regression lines are parallel to the x-axis or upward to the right, and the variance is large.

In a 0.3 mm crack, RM changed from 33.5% at day 7 to 52.7% on day 28. The healing rate of BM starts from 43.1% and increases to 85% on day 28. In the case of EPM, the healing rate reached 63.7% on day 7 and 81.2% on day 28. BPM started at 77.2% on day 7 and reached 96.1% on day 28.

**Figure 12:**
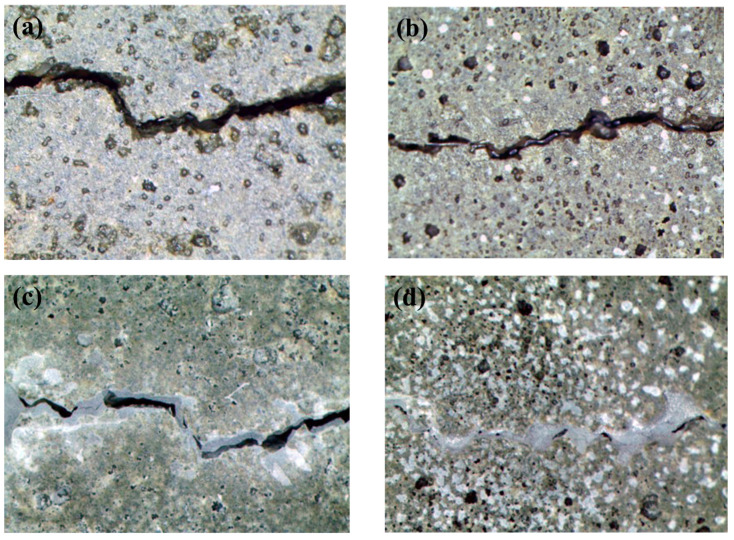
Visual crack closure (**a**) BM at day 0, (**b**) BPM at day 0, (**c**) BM at day 28 and (**d**) BPM at day 28.

Figure 12 shows a crack photograph of a specimen subjected to a water penetration test. Figure 12a,b are BM and BPM of 0 days, respectively, showing that white crystals were formed on the surface of the specimen and near cracks as in figures c,d after 28 days. However, figure d covers most of the cracks compared to figure c.

## 4. Discussion

### 4.1. Effects of MC-Based Pellet on Bacterial Activity

As mentioned in the introduction, methylcellulose-based bacterial pellets are able to enhance MICP. First, as inferred from the bacterial survival experiment that was conducted, the survival rate inside the cement mortar was improved as the number of bacteria at 28 days increased by 8.45 times in BPM compared to BM. Although the bacterial survival of the BM specimen is higher than that of conventional non-cocultured sporulated bacteria [23,38], the long-term bacterial viability can be increased when using MC pellets. The higher survival rate inside the cement composites is known to affect the growth rate of bacteria, the amount of EPS formation, and the calcium carbonate precipitation rate when concrete cracks occur [46], which is thought to influence the bacterial activity, as shown in Figure 6. In the fluorescence-staining experiment, the distribution of bacteria can be observed. In the case of BM, the nutrients are released from the cement paste, which supplies DIC so that the activity of the bacteria (the green, fluorescent area) can be confirmed in the area except for the aggregate [37]. Thus, this may mean that the portion of the aggregate other than the paste does not provide an appropriate biological anchor or nucleation site. In contrast, in the case of BPM, the active distribution of the bacteria can be observed throughout the entire area of the specimen. This is because the MC pellet effectively provided the nutrients, DIC, and Ca^2+^ sources, as well as an appropriate biological anchor to the entire surface of the cement composite, including the aggregate area. As a result, transparent calcium carbonate formed on the surface layer of the specimen, and a cluster of bacteria glowing in fluorescence under the crystal, could be confirmed. 

### 4.2. Effects of MC-Based Pellet on Self-Healing of the Cement Mortar

The contribution of the MC pellet to self-healing capacity of the concrete is divided into three mechanisms. First, pellets with a relatively low strength compared to the aggregates and paste could change the crack characteristics [47]. As shown in Figure 9, in the case of RM and BM, which have no pellet inside, the Q_0_ values measured immediately after crack-inducing specimens are approximately around 1.8–2.1 mL/min·mm at 0.3 mm crack width, whereas the EPM and BPM, which contain pellets, are that of the range of 1.6–1.7 mL/min·mm. Hence, when the pellets are incorporated into the cement composite, the crack environment changes into a condition in which it is difficult for the liquid to flow through a narrow channel, which can be interpreted as reducing the inflow of liquid into the cracked cement composite [48]. Nevertheless, as described later, this phenomenon is also partially related to the strength reduction of the cement composite and it cannot be determined whether it has a positive or negative effect.

The second is the crack-filling effect caused by pellet expansion. As shown in Figure 5a, the pellet expands up to 2.6 times in 24 h when it comes into contact with water. As a result, crack healing by the water permeability test increased by 81% in 28 days, as shown in Figure 11c. However, owing to the characteristics of the pellets, which are composed of organic matter and some inorganic powders, it is believed that this healing effect did not last or proceed further, as shown in Figure 11c. The healing effect sharply declines after 14 days.

Lastly, it is due to the MICP augmented by the pellets mentioned in the previous section. While EPM could not confirm a distinct decrease in water permeability after 14 days, BPM showed a high healing rate of 77.2% in 7 days and showed more than 90% healing on day 14. This accelerated crack healing rate is thought to be due to the combination of the expansion of the pellet and the increased bacterial activity by the pellet.

### 4.3. Effects of MC-Based Pellet on Mechanical Properties of the Cement Mortar

Although the use of bacterial pellets had a positive effect on bacterial activity and self-healing capability, negative effects were also confirmed. First, the initial flow of fresh mortar decreased by 18% compared with that of RM, and the flow loss up to 60 min was 35.7% compared with the RM, at 13.1%. It is presumed that even at high pH, the pellet, which will expand in a smaller amount than in neutral solution, absorbs water around the cement composite, resulting in a decrease in its workability. In addition, it can be seen that the difference in flow is relatively large compared to the difference in strength between BPM and EPM, which is presumed to be due to the acidic yeast extract. Yeast extract is mixed in the same amount in BPM and EPM, but a part of the yeast extract is consumed by some bacteria in BPM, and for this reason, BPM has a more basic environment than EPM. In other words, it is suggested that the EPM absorbed the free water of the initial mortar and caused more severe flow loss. In addition, the flow of BM is higher than that of RM because small pores generated by bacterial metabolism, caused by contact between bacteria and nutrients, increase the flowability, as shown by Jang et al. [38].

Both BM and BPM, which contain bacteria, showed a decrease in strength. An average of 11.59% strength reduction of BPM and EPM appeared in the 28-day curing RM specimen. Strength reduction due to pellet incorporation reduces the relatively weak strength of the pellet compared to the conventional aggregate. As stated by Jang et al. (2020), BM showed a severe decrease in strength of approximately 27.1% of the RM at 28 days of curing, probably due to the creation of micropores by the bacteria [38]. However, in the case of BM, there is little room for improvement, as the entire microstructure of the cement composite is changed by the bacteria, whereas in the case of BPM, the influence of the pellets is related to the strength and adhesion of the pellet to the composite, so there is room for improvement in strength and flow by adjusting the pellets later.

## 5. Conclusions

In this study, the methylcellulose-based bacterial pelletizing technique was used to promote the self-healing of cement composites by bacteria.

- Pellets increased the survival rate of bacterial spores in the mortar by about 3.5 times in 28 days.
- Fluorescence imaging showed that the use of pellets enhanced bacterial activity after the cracks’ cross-section contact with the water
- crack permeability decreased by 90% or more within 14 days when the bacterial pellet was incorporated into the mortar, compared with the specimen containing only the bacteria for 28 days or more.
- However, the workability of the fresh mortar was adversely affected, and the 28-day compressive strength was degraded by 10% compared to the reference mortar.

In conclusion, MC-based pellets are expected to harbor much opportunity for future development of crack self-healing concrete using MICP bacteria. Mortar incorporated with MC pellets healed the crack quickly and effectively, despite its bacterial density being 94% lower than the mortar with bacteria powder only. Considering that the biggest problems in the practical use of bacterial self-healing concrete are feasibility and the fact that bacterial production cost is significantly higher than that of existing construction materials, the use of MC pellets will be an appropriate alternative. Future research on pellet optimization and coating techniques is expected to produce economical and effective bacterial self-healing concrete without the loss of workability and strength.

## Figures and Tables

**Table 1:** Pellet materials and their mixing ratios.

Components	Constituents	CAS No. (Manufacturer)	Mixing Ratio by Weight	
			Bacterial Pellet (BP)	Empty Pellet (EP)
Binder	Methylcellulose (MC)	9004-67-5 (Junsei)	1.0	1.0
	Microcrystalline cellulose (MCC)	9004-34-6 (Daejung)	0.1	0.1
Filler	Silicon dioxide (SiO_2_)	7631-86-9 (Samchun)	3.0	3.0
	Diatomaceous earth (DE)	68855-54-9 (Celite)	0.485	0.4
Active substances	Cocultured bacteria	*L. boronitolerans* YS11 *Bacillus* sp. AK13	0.025	-
	Calcium lactate pentahydrate	5743-47-5 (Duksan)	0.05	0.05
	Yeast extract	8013-01-2 (Samchun)	0.05	0.05

**Table 2:** Mixture proportions for the mortar by cement weight ratio.

Mixture Title *	Cement	Water	Sand	Bacterial Mix **	Empty Pellet (EP)	Bacterial Pellet (BP)
RM	1.0	0.4	2.0	-	-	-
BM				0.03	-	-
EPM				-	0.05	-
BPM				-	-	0.05

* RM: Reference mortar, NM: Bacteria spore mortar, EPM: Empty pellet mortar, BPM: Bacterial pellet mortar. ** Bacterial mix: mixture of cocultured bacteria and their nutrients.
